# Comment on “Modulation of Metabolic Detoxification Pathways Using Foods and Food-Derived Components: A Scientific Review with Clinical Application”

**DOI:** 10.1155/2015/934070

**Published:** 2015-10-12

**Authors:** Tanis R. Fenton, Beth Armour, Jayne Thirsk

**Affiliations:** ^1^PEN, Dietitians of Canada, Canada; ^2^Department of Community Health Sciences, Alberta Children's Hospital Research Institute, O'Brien Institute for Public Health, Cumming School of Medicine, University of Calgary, Calgary, AB, Canada T2N 4Z6

The article “Modulation of Metabolic Detoxification Pathways Using Foods and Food-Derived Components: A Scientific Review with Clinical Application” is misleading in its title and the discussion does not fit this title. This narrative review article presents a comprehensive review of food and food components on specific metabolic detoxification pathways (phase I cytochrome enzymes, phase II conjugation enzymes, antioxidant support systems, and metallothionein) for naturopathic therapies in 11 extensive tables.

This paper has many strengths; the strongest of these is the authors' discussion of the important limitations of this science. While their emphasis was about finding evidence to support clinical recommendations to use foods and food-based constituents to reduce toxins, they recognized the following.

“It is best to take precaution in firmly advocating foods or food-based nutrients that only have cell or animal data as support. It is best to rely on the clinical [human] studies”; “science has not fully demonstrated the individual impacts of these [smoking, physical activity, or stress] factors, along with all of them together to be able to understand the effects of altering the function of some detoxifying enzymes”; “in several instances, certain foods exhibited a particular activity on an enzyme, while, at higher doses, they had another, opposite effect”; “for patients who are taking multiple pharmaceuticals, it is important to know which detoxification systems will be influenced by nutrients and foods so that side effects are minimized or avoided”; “without a full understanding of a patient's SNPs [single nucleotide polymorphisms], it becomes difficult to make accurate assessments about nutrients and dosing”; “in some of the research presented here, effects on detoxification enzymes were seen after several days of food intake or supplementation, while, in other cases, induction of an enzyme might be fairly rapid, followed by efficient adaptability” [1].

Given all of the limitations to current knowledge and the large effects seen in a few sparse studies of food-based compounds altering detoxification enzymes and the metabolism of medications [2–5] and other compounds such as steroid hormones [6] and selected carcinogens [7], the paper's title, which states that the authors have produced “clinical applications,” is misleading. The paper does not provide clinical applications. The tables in the paper do not indicate the direction of effects of the tested foods/food-based components and which outcomes the test substance was assessed for. We agree with the authors' conclusion about the state of knowledge: “the resulting clinical takeaway might be to encourage patients to follow a mixed, varied diet, full of different plant-based, whole foods” [1].

More research is needed before knowledge is sufficient to be able to provide advice to individuals on how to raise their detoxifying abilities. This science is in its infancy, as this review capably pointed out.
